# A bibliometric and visual analysis of cancer-associated fibroblasts

**DOI:** 10.3389/fimmu.2023.1323115

**Published:** 2023-12-19

**Authors:** Wei-Chen Yuan, Jie-Xiang Zhang, Hai-Bin Chen, Ying Yuan, Yu-Pei Zhuang, Hong-Li Zhou, Mu-Han Li, Wen-Li Qiu, Hong-Guang Zhou

**Affiliations:** ^1^ Department of Oncology, Affiliated Hospital of Nanjing University of Chinese Medicine, Nanjing, China; ^2^ Jiangsu Collaborative Innovation Center of Traditional Chinese Medicine in Prevention and Treatment of Tumor, The First Clinical College of Nanjing University of Chinese Medicine, Nanjing, China; ^3^ The First Clinical College, Shandong University of Traditional Chinese Medicine, Jinan, China; ^4^ Science and Technology Department, Jiangsu Collaborative Innovation Center of Traditional Chinese Medicine Prevention and Treatment of Tumor, Nanjing University of Chinese Medicine, Nanjing, China; ^5^ Department of Otorhinolaryngology, Oral Plastic Surgery, Affiliated Hospital of Weifang Medical College, Weifang, China; ^6^ College of Pharmacy, Nanjing University of Chinese Medicine, Nanjing, China; ^7^ Department of Radiology, Affiliated Hospital of Nanjing University of Chinese Medicine, Nanjing, China

**Keywords:** cancer-associated fibroblasts, cancer, bibliometrics, research trends, hot spots

## Abstract

**Background:**

Cancer-associated fibroblasts (CAFs) represent the predominant stromal component within the tumour microenvironment (TME), exhibiting considerable heterogeneity and plasticity that significantly impact immune response and metabolic reprogramming within the TME, thereby influencing tumour progression. Consequently, investigating CAFs is of utmost importance. The objective of this study is to employ bibliometric analysis in order to evaluate the current state of research on CAFs and predict future areas of research and emerging trends.

**Methods:**

Conduct a comprehensive search for scholarly publications within the Web of Science Core Collection database, encompassing the time period from January 1, 2001, to December 31, 2022. Apply VOSviewer, CiteSpace, R software and Microsoft Excel for bibliometric analysis and visualisation.

**Results:**

This study involved a comprehensive analysis of 5,925 publications authored by 33,628 individuals affiliated with 4,978 institutions across 79 countries/regions. These publications were published in 908 journals, covering 14,495 keywords and 203,947 references. Notably, there was a significant increase in articles published between 2019 and 2022. China had the highest count of articles, while the United States emerged as the most frequently cited country. The primary research institutions in this field were Shanghai Jiao Tong University, Harvard University, and the University of Texas MD Anderson Cancer Center. Sotgia, Federica and Lisanti, Michael P from the University of Manchester, and Martinet, Wim from the University of Antwerp were the most prolific and highly cited authors. The journal Cancers had the highest number of publications, while Cancer Research was the most frequently cited journal. Molecular, biology, immunology, medicine and genetics were the main research disciplines in the field of CAFs. Key directions in CAFs research encompassed the study of transforming growth factor-β, Fibroblast Activation Protein, breast cancer, as well as growth and metastasis. The findings from the analysis of keyword co-occurrence and literature co-citation have revealed several emerging hotspots and trends within the field of CAFs. These include STAT3, multidrug resistance, pancreatic ductal adenocarcinoma, pan-cancer analysis, preclinical evaluation, ionizing radiation, and gold nanoparticles.

**Conclusion:**

Targeting CAFs is anticipated to be a novel and effective strategy for cancer treatment. This study provides a comprehensive overview of the existing research on CAFs from 2001 to 2022, utilizing bibliometric analysis. The study identified the prominent areas of investigation and anticipated future research directions, with the aim of providing valuable insights and recommendations for future studies in the field of CAFs.

## Introduction

Resting mesenchymal cells, commonly known as fibroblasts, are found in normal tissues, residing within the extracellular matrix (ECM) composed of interstitial fibers. These fibroblasts undergo activation in response to environmental factors, particularly during processes such as wound healing, tissue inflammation, and organ fibrosis (1). In contrast, fibroblasts in tumour tissue transform into cancer-associated fibroblasts (CAFs) by receiving signals from nearby tumourigenic cells. CAFs are the predominant stromal cells within the tumour microenvironment (TME). They possess the ability to secrete a diverse range of cytokines and metabolites, thereby impeding immune cell functionality and fostering the advancement of tumours, invasion, angiogenesis, therapeutic resistance, and immune evasion (2–6). As a result, CAFs have emerged as a significant focus in the field of cancer treatment.

In response to tumour-derived stimuli, CAFs are primarily generated by fibroblasts and/or stellate cells residing in the pancreas and hepatic, but CAFs may also arise from bone marrow mesenchymal stem cells that are recruited to the tumour or undergo transformation from adipocytes, pericytes, and endothelial cells (7). Fibroblasts can serve as biomarkers indicating the presence of an immunosuppressive TME, while CAFs contribute to tumour progression by inducing immunosuppression (8). On the one hand, CAFs can establish and modify the ECM framework, thereby facilitating the invasion of tumour cells within the TME. Additionally, CAFs can secrete growth factors, cytokines, and chemokines, which interact with cancer cells or other stromal cells, ultimately impeding the activity of immune cells (9). For example, CAFs secrete transforming growth factor-β (TGF-β), stromal-derived factor 1 (SDF-1), platelet-derived growth factor (PDGF), vascular endothelial growth factor (VEGF) and interleukins (IL), which generate tumour vasculature, promote immunosuppression and enhance drug resistance (10). Furthermore, CAFs secret IL-6 and C-X-C motif chemokine ligand 2 (CXCL12) to strengthen the population of regulatory T cells within the TME and activate factor-related apoptosis ligand and programmed death receptor ligand-2 to induce cytotoxic T cells death in an antigen-dependent manner, thereby inhibiting anti-tumour immunity and immunotherapy (11). On the other hand, CAFs secret large amounts of collagen, fibronectin, etc., to promote the solidification of the extra-tumoural stroma, and the ECM is more abundant, concentrated, and hard in the tumour than in the surrounding healthy tissues, which leads to an increase in the pressure of the interstitial fluid and makes it difficult for the tumour to obtain nutrients, oxygen, immune cells, and therapeutic drugs (12). Moreover, CAFs have the ability to induce Epithelial to mesenchymal transition (EMT), along with aerobic glycolysis, to produce more metabolites such as pyruvate, lactate, and fatty acids, which promote tumour metabolism and nourishment of cancer cells and affect metabolic reprogramming of tumours (13).

CAFs exhibit considerable heterogeneity and plasticity, giving rise to various subtypes that may exert opposing cellular functions (3). α-smooth muscle actin (α-SMA), S100 calcium binding protein A4/fibroblast specific protein 1, fibroblast activation protein (FAP), and Desmin are widely recognized as prevalent activation markers of CAFs (14). Markers for various subtypes of CAFs exhibit variability. Upon receiving diverse signals from other tumour components, such as cytokines, chemokines, or growth factors, stromal cells can generate heterogeneous populations of CAFs with distinct phenotypes and functions. These functions extend beyond the conventional tumour-promoting role (15) and may encompass diverse tumour-suppressive functions. Recently, there has been a notable increase in the volume of publications pertaining to research on CAFs. However, the intricate and uncertain mechanism of action exhibited by heterogeneous subpopulations of CAFs poses a significant challenge in the field of precision tumour therapy. Consequently, considering their intricate involvement in tumour therapy, it is crucial to conduct a quantitative assessment of the current state, research focus areas, and future directions regarding CAFs. Bibliometric analysis, an interdisciplinary field that combines mathematics, statistics, and bibliography, is employed to quantify and visualize research trends. This method has emerged as a crucial approach in assessing the quality and impact of scholarly works (16, 17). The objective of this study is to investigate the research profile of CAFs with the aim of generating novel insights and potential avenues for future research within the domain of CAFs.

## Methods

### Search strategies

In this research, the Web of Science Core Collection (WOSCC) database was utilized as a primary data origin. To ensure the credibility and accuracy of the findings, we used “cancer-associated fibroblasts” as the designated search term and improved the search method by incorporating insights from previous studies (16, 18). Detailed search formulas are available in the **Supplementary Material**. The publication period covered a span of 22 years, starting from January 1, 2001, and ending on December 31, 2022. The articles included in this study are categorized as either Article or Review Article and are written in the English language. To mitigate potential systematic bias resulting from database updates, we conducted a comprehensive search and screening of publications, which was completed on September 23, 2023.

### Data analysis

We applied Figdraw (www.figdraw.com) to create flowcharts and Microsoft Excel 2021 to create statistical tables and trend graphs. This study employed the bibliometrix 4.1.3 tool in R 4.3.1 software to conduct Lotka’s Law analysis and heat mapping. Furthermore, the bibliometric analyses of countries/regions, institutions, authors, journals, keywords, and references were conducted using VOSviewer 1.6.19 and Citespace 6.2 R4. The main emphasis was placed on examining co-authorship, co-occurrence, and co-citation patterns. We consolidated overlapping items into a unified element, rectified misspelt words through artificial means, and conducted data cleaning before exporting the data for subsequent analysis.

## Results

**Figure 1:**
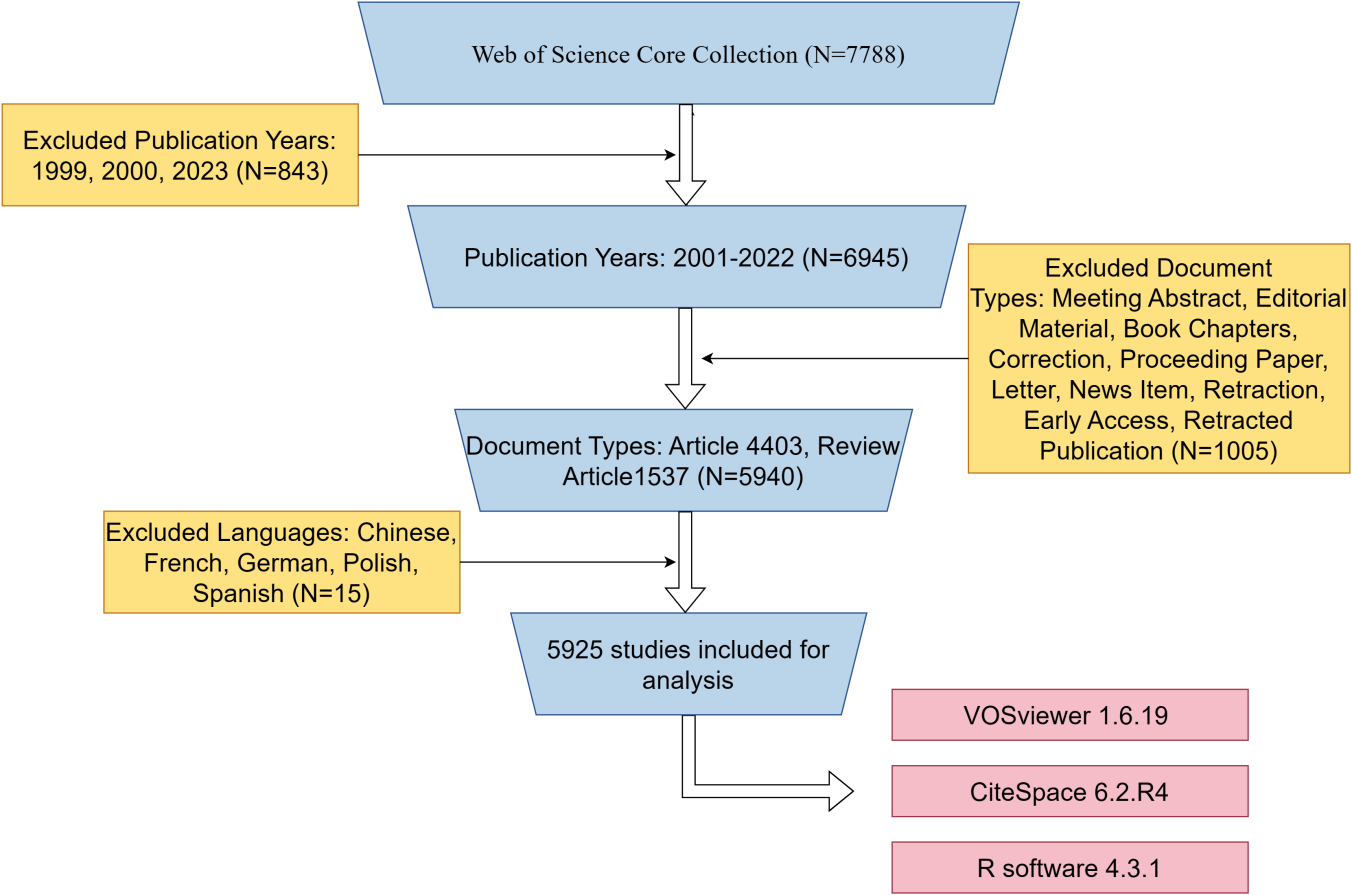
Flowchart of the search strategy and exclusion criteria.

We retrieved a total of 7788 published papers from WOSCC, set up exclusion criteria based on publication time, article type, and publication language, and finally screened 5925 papers for bibliometric analysis. The specific flow chart was shown in **Figure 1**.

### Trends in the growth of publications

**Figure 2:**
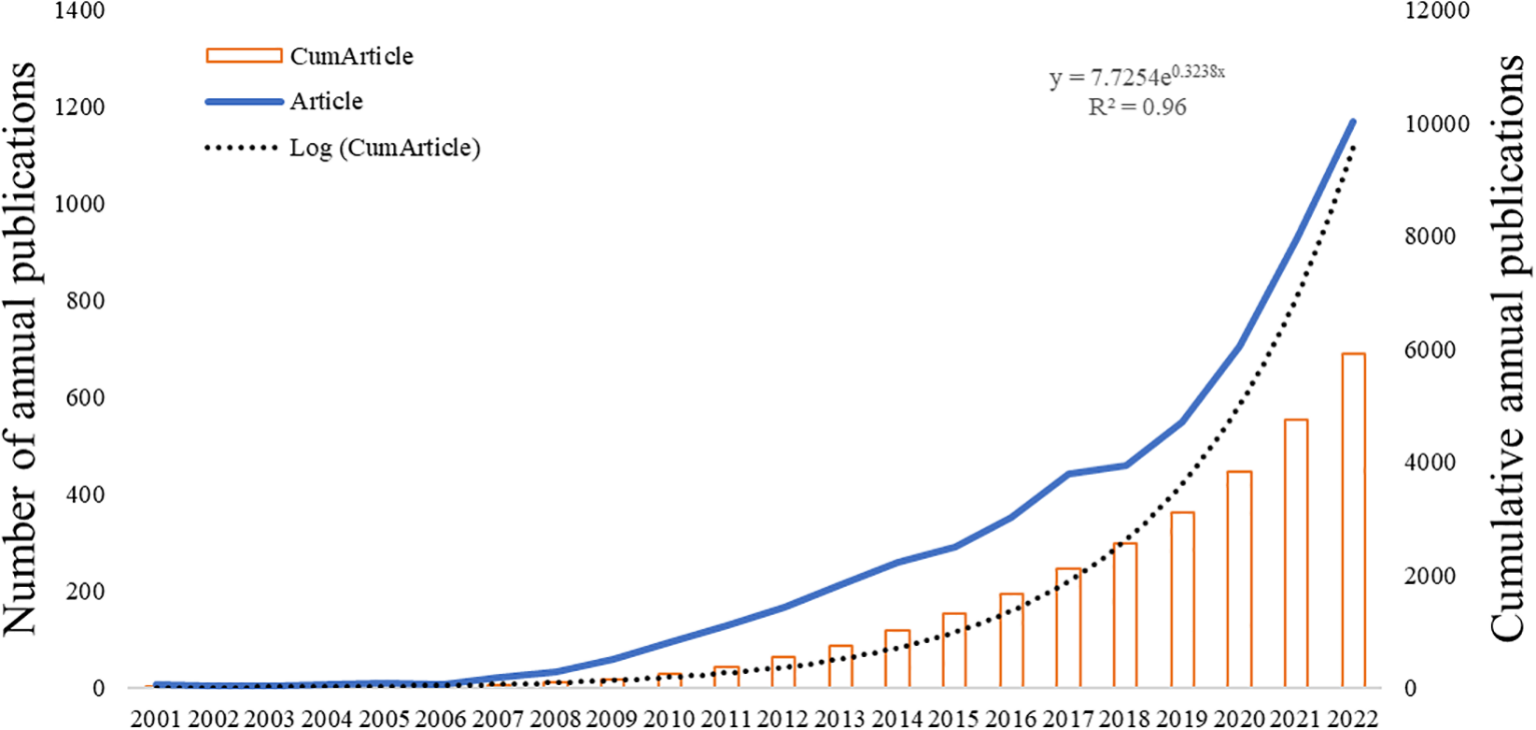
Trend graph of the growth of annual and cumulative annual number of CAFs’ publications.

The overall growth trend in the annual number of articles published by CAFs was shown in **Figure 2**, which showed that the number of published articles was increasing year by year from 2001 to 2022. The output of articles from 2001 to 2006 exhibited a low count, with an annual average of fewer than 20 articles. However, from 2007 to 2018, there was a consistent and notable increase in the number of articles published, resulting in a cumulative total of 2,525 articles. This figure accounts for approximately 42.6% of the total articles published within the broader timeframe of 2001 to 2022. During the period from 2019 to 2022, there was a notable rapid growth in the number of publications, with over 500 publications in 2019 and an even larger number exceeding 1,000 publications in 2022. This indicates that CAFs have garnered increasing attention and emerged as a popular research direction, entering a phase of rapid development.

### Situation of countries/regions and institutions

**Figure 3:**
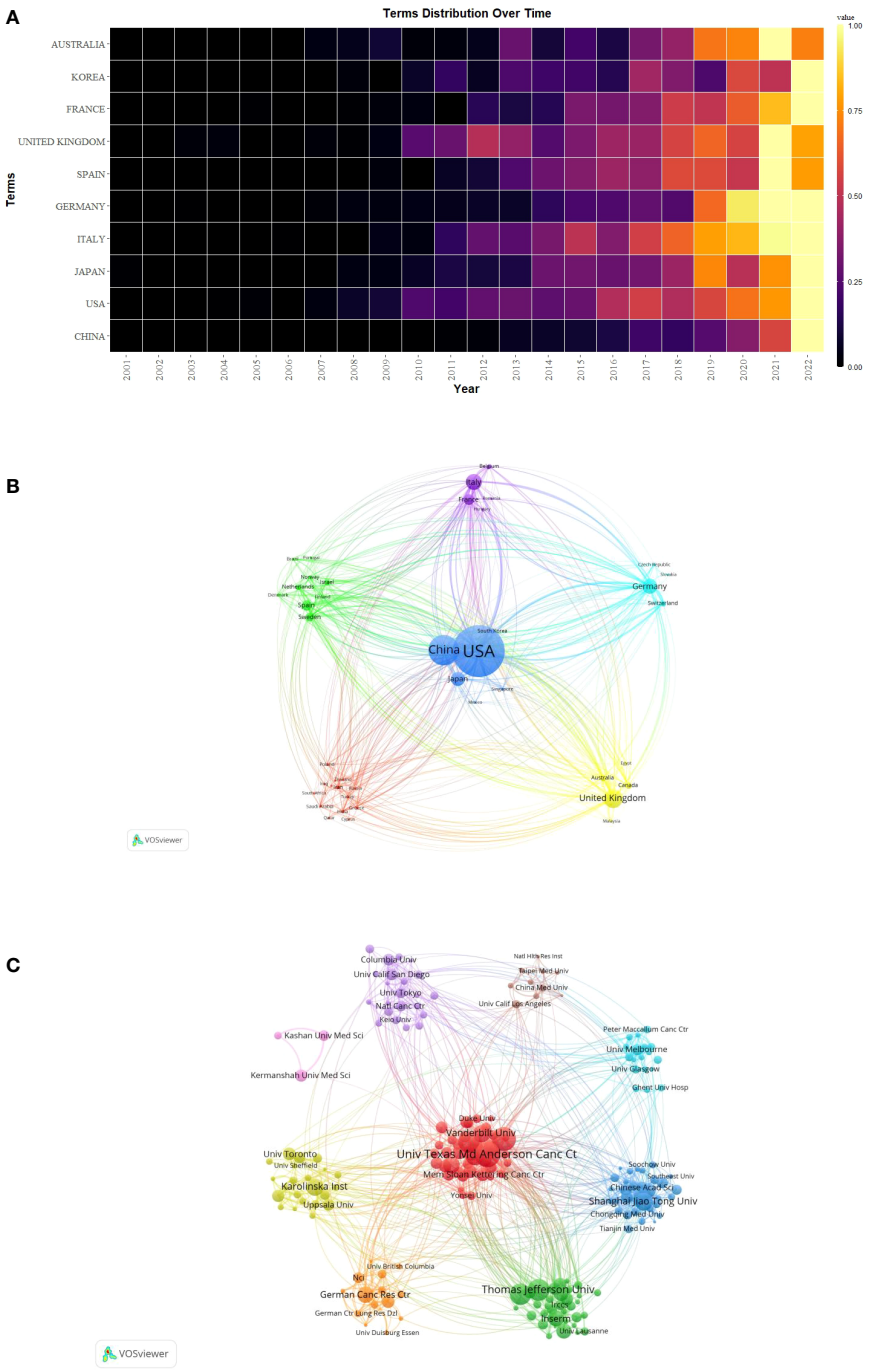
**(A)** Heat map of annual postings for the top 10 countries/regions. **(B)** Network map of countries/regions for CAFs studies. **(C)** Institutional network map for CAFs studies.

The publication of CAFs was carried out by a total of 4,978 institutions across 79 countries/regions, as indicated in **Table 1**, which presented the top 10 countries/regions and institutions in terms of productivity. China emerged as the leading contributor with 1,860 papers published, followed by the United States of America (USA) with 1,642 papers and Japan with 596 papers. In terms of citations received, the USA garnered the highest count with 124,387 citations, followed by China with 60,934 citations and the United Kingdom with 31,735 citations. Although China has the highest number of publications, the average number of citations per paper (N=32.76) is much lower than USA (N=75.75) and Japan (N=78.94) and lower than Sweden (N=80.36) and France (N=77.09), which suggests that Chinese scholars’ articles have a low academic impact, and they still need to publish further academic papers of higher quality, more innovative and widely recognized in their professional fields. During the study period, there was a notable increase in annual citations per publication, particularly in 2021-2022, across most countries/regions (**Figure 3A**).

**Table 1:** The top 10 countries/regions and institutions for CAFs publications.

Country	Count	Centrality	Citation	Institution	Count	Centrality	Citation
USA	1642	0.42	124387	Univ Texas Md Anderson Canc Ctr	127	0.04	14169
CHINA	1860	0.06	60934	Harvard Univ	131	0.03	12361
UNITED KINGDOM	402	0.14	31735	Thomas Jefferson Univ	85	0.02	10273
ITALY	422	0.16	26267	Univ Manchester	72	0.02	9865
GERMANY	392	0.15	24090	Mit	43	0.02	9361
JAPAN	596	0.14	21687	Univ Calif San Francisco	36	0.02	8175
FRANCE	206	0.25	15880	Shanghai Jiao Tong Univ	135	0.01	6758
SPAIN	216	0.05	14928	Vanderbilt Univ	55	0.02	6478
SWEDEN	145	0.04	11652	Karolinska Inst	96	0.03	6196
AUSTRALIA	165	0.03	8265	Cold Spring Harbor Lab	18	0.01	6078

Additionally, we conducted a network mapping analysis to identify and illustrate the robust communication patterns observed between countries/regions (**Figure 3B**). The size of the node’s centrality represents the closeness of communications between countries/regions, with a general centrality greater than 0.1 representing a denser degree of connectivity and a higher degree of relatedness. Among them, the USA centrality was as high as 0.42, which indicated that it acted as a bridge for cooperation and exchange of papers among countries. In addition, France (N=0.25), Italy (N=0.16), Germany (N=0.15), United Kingdom (N=0.14), and Japan (N=0.14) were all significant nodes in the cluster, representing a high degree of cooperation.

The 4,978 institutions formed nine major clusters (**Figure 3C**). Shanghai Jiao Tong University, Harvard University, and The University of Texas MD Anderson Cancer Center were the institutions with the highest number of publications, 135, 131, and 127, respectively. Also, 7 of the top 10 institutions were located in the USA, which demonstrated the high interest of USA institutions in the study of CAFs and highlighted their important position and contribution to this field of research. The University of Texas MD Anderson Cancer Center received the most citations (N=14169), followed by Harvard University (N=12361) and Thomas Jefferson University (N=10273), both USA institutions. The University of California, San Francisco had the highest average number of citations per paper (N=227.08), followed by Massachusetts Institute of Technology (N=217.70) and the University of Manchester (N=137.01). As none of the centrality of the individual agency nodes exceeded 0.1, the centrality ranged from 0.01 to 0.04, suggesting that the degree of cooperation between international agencies was relatively at low level.

### Authors and co-authors analysis

**Figure 4:**
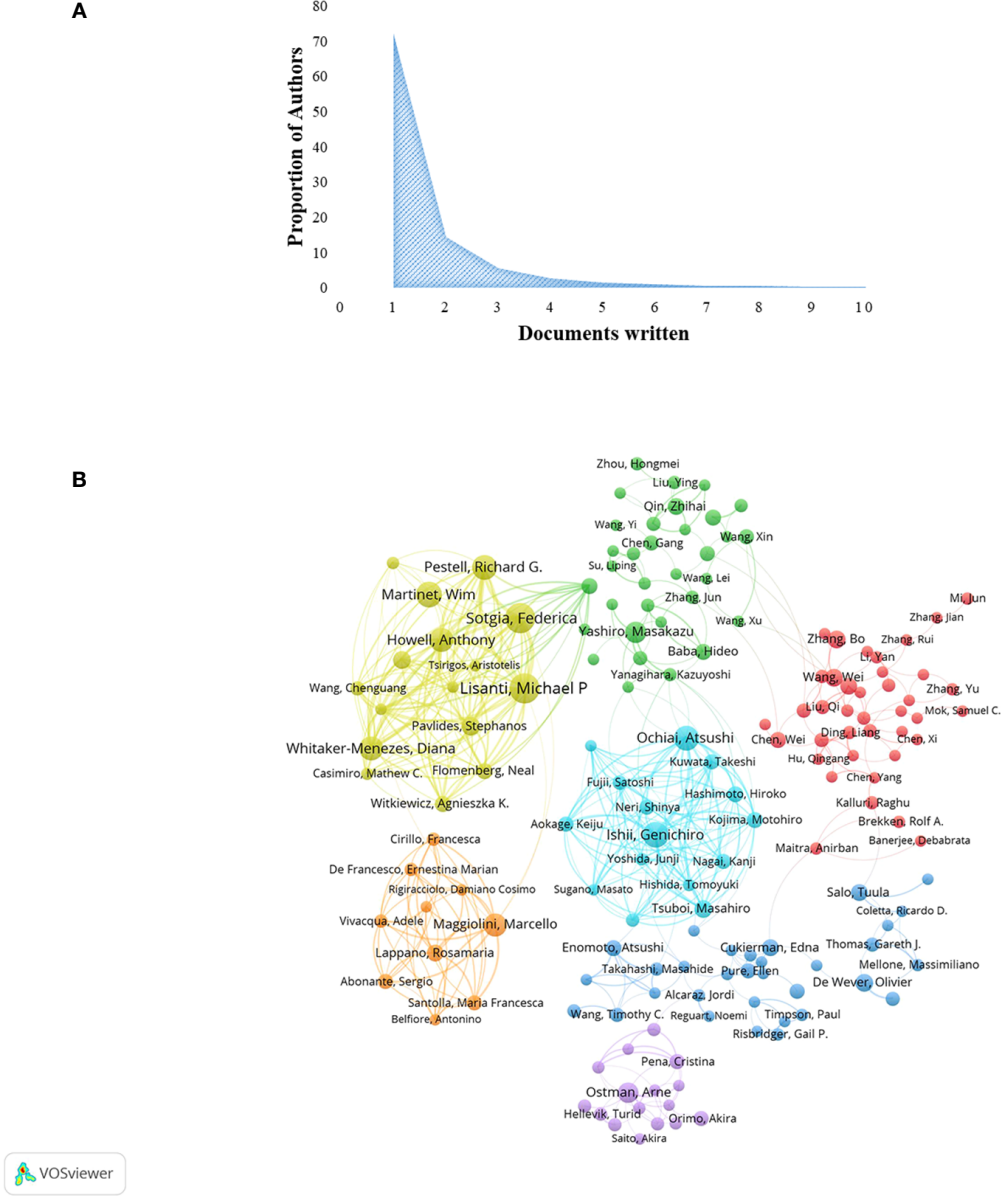
**(A)** Scientific productivity of authors based on Lotka’s law. **(B)** Network diagram of author collaborations for CAFs studies.

A total of 33,628 authors were involved in the study of CAFs. The analysis of scientific productivity, using Lotka’s law, revealed that the majority of authors, accounting for 72.10%, had published only one paper (**Figure 4A**). A smaller proportion, 14.40%, had published two papers, while a mere 5.70% had published three papers. Furthermore, **Table 2** presented the top 10 authors who demonstrated high productivity and citation rates within the domain of CAFs. Sotgia, Federica from the University of Manchester published the most papers (N=59), followed by Martinet, Wim from the University of Antwerp (N=51) and Lisanti, Michael P from the University of Manchester (N=50). The authors with the highest number of citations at the same time were also the three mentioned above, although in a different order from the prolific authors, Lisanti, Michael P (N=8772), Sotgia, Federica (N=8731) and Martinet, Wim (N=7179). In addition, the H-index of the three authors mentioned above was at a high value: Lisanti, Michael P (N=136), Sotgia, Federica (N=85) and Martinet, Wim (N=89). It could be seen that they had an important position in the field of CAFs research, made significant contributions, and were the leaders in the field of CAFs. Furthermore, Pestell, Richard G. from Wistar Cancer Center (43 publications, 7138 citations, H-index 121), Howell, Anthony from Wythenshawe Hospital (40 publications, 5637 citations, H-index 112) and Kalluri, Raghu from UTMD Anderson Cancer Center (12 publications, 6266 citations, H-index 115) were also important researchers in the field of CAFs. We additionally found positive cooperation between clusters of authors of seven different colours (**Figure 4B**). There existed a level of collaboration between two interconnected nodes situated in distinct clusters, namely Lisanti, Michael P, Maggiolini, Marcello, and Su, Liping.

**Table 2:** The top 10 most prolific and cited authors in the field of CAFs.

Author	Count	H-index	Cited Author	Count	H-index
Sotgia, Federica	59	85	Lisanti, Michael P	8772	136
Martinet, Wim	51	89	Sotgia, Federica	8731	85
Lisanti, Michael P	50	136	Martinet, Wim	7179	89
Ochiai, Atsushi	43	74	Pestell, Richard G.	7138	121
Pestell, Richard G.	42	121	Whitaker-Menezes, Diana	6482	48
Howell, Anthony	40	112	Kalluri, Raghu	6266	115
Maggiolini, Marcello	37	56	Howell, Anthony	5637	112
Whitaker-Menezes, Diana	37	48	Pavlides, Stephanos	5190	37
Chiarugi, Paola	36	64	Tuveson, David A.	5142	75
Yashiro, Masakazu	35	60	Flomenberg, Neal	4405	55

### Journals and cited academic journals

**Figure 5:**
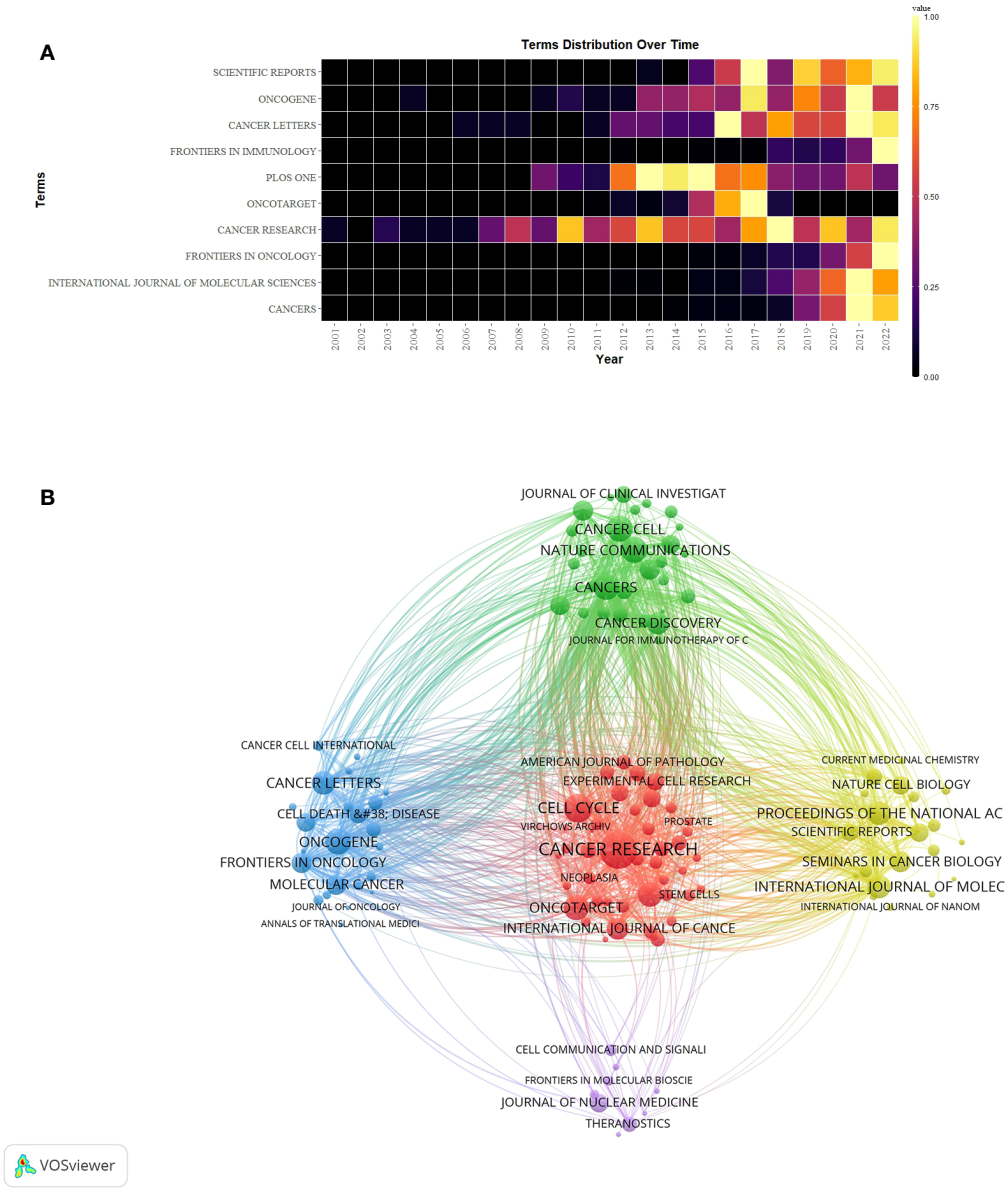
**(A)** Heat map of annual publications in the top 10 journals. **(B)** Network diagram of journals for CAFs studies.

A grand total of 908 scholarly journals actively engaged in conducting research on CAFs. **Table 3** showed the top 10 journals in terms of number of publications and cited journals related to the field of CAFs. The journal with the most publications was Cancers (N=316), followed by International Journal of Molecular Sciences (N=156) and Frontiers in Oncology (N=153). The top 10 journals in terms of number of publications included 5 journals with Q1 Journal Citation Reports (JCR) partitioning, 4 journals with Q2 JCR partitioning, and 7 journals with impact factors (IF) greater than 5 points. Cancer Research emerged as the journal with the highest number of citations (N=14997) among the top 10 cited journals, succeeded by Cell Cycle (N=8247) and Nature Communications (N=6593). There were 7 journals with Q1 JCR partitioning, 2 with Q2 JCR partitioning, and 8 with impact factors greater than 5 points. Simultaneously, the study period witnessed a notable surge in the annual publication count across numerous journals, particularly in 2021-2022 (**Figure 5A**). Notably, Plos One, Oncotarget, Scientific Reports, and Cancer Research have established a strong presence in the field of CAFs, with their publication volume reaching its peak between 2013 and 2018.

**Table 3:** The top 10 issued and cited journals for CAFs studies.

Journal	Count	JCR (2022)	IF (2022)	Cited Journal	Citation	JCR (2022)	IF (2022)
CANCERS	316	Q1	6.575	CANCER RESEARCH	14997	Q1	13.312
INTERNATIONAL JOURNAL OF MOLECULAR SCIENCES	156	Q2	6.208	CELL CYCLE	8247	Q2	5.173
FRONTIERS IN ONCOLOGY	153	Q2	5.738	NATURE COMMUNICATIONS	6593	Q1	17.694
CANCER RESEARCH	144	Q1	13.312	CANCER CELL	6487	Q1	38.585
ONCOTARGET	141	–	–	CANCERS	6481	Q1	6.575
PLOS ONE	120	Q2	3.752	ONCOGENE	6314	Q1	8.756
FRONTIERS IN IMMUNOLOGY	111	Q1	8.786	ONCOTARGET	6072	–	–
CANCER LETTERS	93	Q1	9.756	PLOS ONE	5610	Q2	3.752
ONCOGENE	93	Q1	8.756	CELL	5058	Q1	66.85
SCIENTIFIC REPORTS	93	Q2	4.996	INTERNATIONAL JOURNAL OF MOLECULAR SCIENCES	5002	Q1	6.208

The network graph of cited journals (**Figure 5B**) represented the degree of association between journals. The cluster of journals was divided into five clusters, with the size of the nodes representing the number of co-citations and the thickness of the connections representing the strength of the association. Journals of the same colour have similar themes to each other, most represented by the red cluster centred on Cancer Research, indicating the strongest degree of thematic connection between journals and the highest number of common citations.

### Analysis of research disciplines

**Figure 6:**
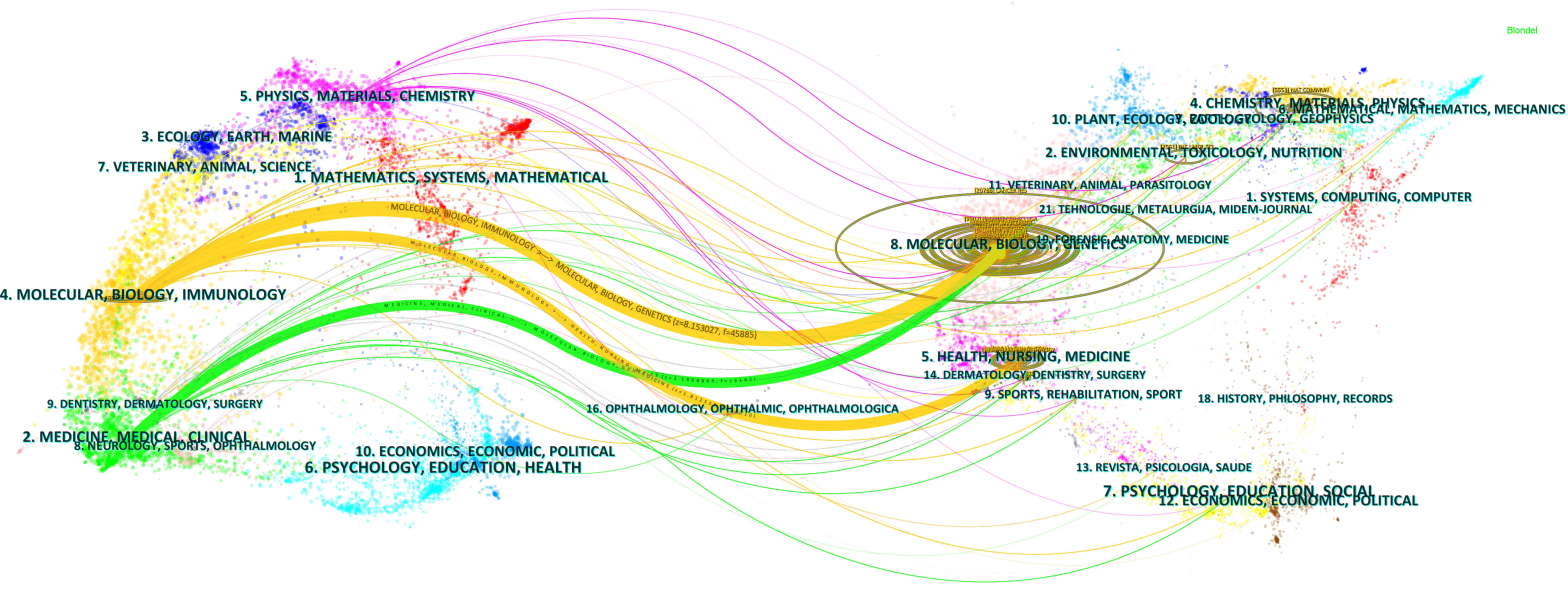
Double image overlay of journals on CAFs studies.

The results of the dual graph overlay of journals can accurately capture the dynamics and trends of disciplinary development. The research on CAFs was found to be related to multiple disciplines, as depicted in **Figure 6**. The visualization graph was divided into two sections: the left part represented sizing journals, reflecting the knowledge frontier, while the right part represented cited journals, reflecting the knowledge base. The curves connecting the left and right sections represent citation links that visualize the collaborative relationships between sizing and cited journals. The trajectories of these links reflect the fluidity of disciplines at the journal level, providing an understanding of the interdisciplinary relationships within the field. The Z-Scores function emphasizes stronger connections, more fluid trajectories, and higher scores, which are represented by thicker linking lines in the visualization. The length of the ellipse indicates the number of authors, while the width of the ellipse represents the number of publications.

As depicted in **Figure 6**, the citing journals were predominantly distributed within discipline 2# (medicine, medical, clinical) and discipline 4# (molecular, biology, immunology). On the other hand, the cited journals are mainly distributed within discipline 5# (health, nursing, medicine) and discipline 8# (molecular, biology, genetics). In particular, publications in the disciplines of molecular, biology, and immunology (yellow track) were significantly influenced by publications in the disciplines of molecular, biology, genetics (z=8.15, f=45885) and health, nursing, medicine (z=1.81, f=11110). In addition, publications in the medicine, medical, and clinical (green track) disciplines were significantly influenced by publications in the molecular, biology, and genetics (z=2.19, f=13185) disciplines.

### References co-citation, clustering, timeline and bursts

**Table 4** listed the top 10 most cited publications in the field of CAFs, among which the study entitled “Stromal fibroblasts present in invasive human breast carcinomas promote tumour growth and angiogenesis through elevated SDF-1/CXCL12 secretion” by Orimo A et al. was the most cited (N=2869). The study indicated that CAFs in breast cancer promoted tumour cell growth by secreting SDF-1, while SDF-1 mediated the entry of endothelial progenitor cells into cancer cells to promote tumour angiogenesis (19). The study was a major experimental breakthrough in the field of breast cancer and CAFs, laying a solid foundation for the subsequent research on the mechanism of CAFs and breast cancer, and influencing the direction of subsequent research.

**Table 4:** The top 10 most cited articles in CAFs studies.

Title	DOI	First Author	Year	Journal	Citations
Stromal fibroblasts present in invasive human breast carcinomas promote tumor growth and angiogenesis through elevated SDF-1/CXCL12 secretion	10.1016/j.cell.2005.02.034	ORIMO A	2005	CELL	2869
The biology and function of fibroblasts in cancer	10.1038/nrc.2016.73	KALLURI R	2016	NAT REV CANCER	2315
Depletion of Carcinoma-Associated Fibroblasts and Fibrosis Induces Immunosuppression and Accelerates Pancreas Cancer with Reduced Survival	10.1016/j.ccr.2014.04.005	OZDEMIR BC	2014	CANCER CELL	1587
A framework for advancing our understanding of cancer-associated fibroblasts	10.1038/s41568-019-0238-1	SAHAI E	2020	NAT REV CANCER	1463
Distinct populations of inflammatory fibroblasts and myofibroblasts in pancreatic cancer	10.1084/jem.20162024	OHLUND D	2017	J EXP MED	1217
Cancer-Associated Fibroblasts Are Activated in Incipient Neoplasia to Orchestrate Tumor-Promoting Inflammation in an NF-κB-Dependent Manner	10.1016/j.ccr.2009.12.041	EREZ N	2010	CANCER CELL	1115
The reverse Warburg effect Aerobic glycolysis in cancer associated fibroblasts and the tumor stroma	10.4161/cc.8.23.10238	PAVLIDES S	2009	CELL CYCLE	967
Mechanotransduction and YAP-dependent matrix remodelling is required for the generation and maintenance of cancer-associated fibroblasts	10.1038/ncb2756	CALVO F	2013	NAT CELL BIOL	888
Pathogenesis, Diagnosis, and Management of Cholangiocarcinoma	10.1053/j.gastro.2013.10.013	RIZVI S	2013	GASTROENTEROLOGY	831
Fibroblast Heterogeneity and Immunosuppressive Environment in Human Breast Cancer	10.1038/s41573-018-0004-1	CHEN XM	2019	NAT REV DRUG DISCOV	822

**Figure 7:**
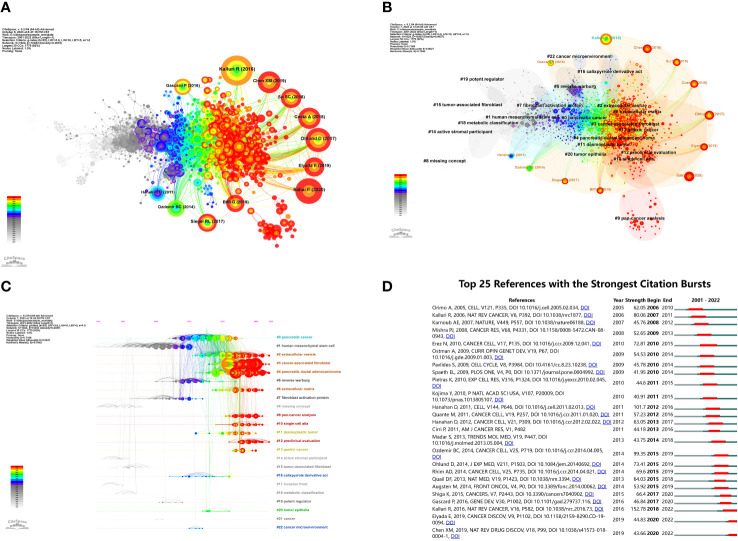
**(A)** Visualization of literature co-citations in CAFs studies. **(B)** Literature co-citations clustering in CAFs studies. **(C)** Timeline plot of literature co-citations clustering in CAFs studies. **(D)** The top 25 references with the strongest citation bursts.

A co-citation analysis was conducted on the literature to elucidate the advancements in research on CAFs. The findings, presented in **Figure 7A**, primarily focused on 12 significant nodes. Notably, a majority of the extensively cited literature emerged from 2011 to 2020, with a particular emphasis on the period between 2017 and 2020. These observations underscore the rapid development and noteworthy outcomes achieved in this field during the specified timeframe.

**Figure 7B** showed the clustering of CAFs co-cited references, suggesting a thematic categorization of the CAFs domain. The figure showed 23 clusters, the first named “#0 pancreatic cancer”, the second named “#1 human mesenchymal stem cell”, and the third named “#2 extracellular vesicle”. The topics of these clusters were all hotspots at various stages in the field of CAFs.

**Figure 7C** showed a timeline view of the clustering of co-cited references. Nodes positioned along the same line in the figure indicated a cluster, and the topic of the cluster was identified by the label # on the right. The larger the node indicated, the higher the co-citation frequency, while from left to right in chronological order, the left node appeared earlier, mostly for classic or relatively outdated topics, and the right node appeared later, mostly for emerging topics. As seen in the figure, several newer hotspots were “#2 extracellular vesicle”, “#3 cancer associated fibroblast”, “#4 pancreatic ductal adenocarcinoma”, “#6 extracellular matrix”, “#9 pan- cancer analysis”, “#10 single-cell atla”, “#12 preclinical evaluation”.

The analysis of bursts of co-cited references facilitated the investigation of the duration of hot content in CAFs. To visually represent this, **Figure 7D** was plotted. The green line in the figure corresponded to the years 2001-2022, while the red line indicated the duration of the burst. Notably, the article “Biology and Function of Fibroblasts in Cancer” (2018-2022, intensity 152.78), authored by KALLURI R in 2016, exhibited the highest burst intensity. This article delved into the physiological properties and functions of fibroblasts in tumours and TMEs, with a specific focus on the functional heterogeneity of fibroblasts (20). Furthermore, the scholarly works that exhibited the highest levels of intensity were “Hallmarks of cancer: the next generation” (2012-2016, intensity 101.7), authored by Hanahan D and “Depletion of Carcinoma-Associated Fibroblasts and Fibrosis Induces Immunosuppression and Accelerates Pancreas Cancer with Reduced Survival” (2015-2019, intensity 99.35) authored by Ozdemir BC.

### Keywords co-occurrence, clusters and bursts

A total of 14,495 keywords were extracted from 5925 papers. As shown in **Table 5**, TGF-β (N=853), FAP (N=254) and nuclear factor kappa-B (NF-κB) (N=237) were the most common molecular keywords. The most frequent keywords for pathological processes were growth (N=1127), metastasis (N=947) and progression (N=733). While breast cancer (N=1144), colorectal cancer (N=682) and pancreatic cancer (N=502) were the diseases most closely associated with CAFs.

**Table 5:** The top 10 molecules, pathological processes and diseases associated with CAFs.

Molecules	Count	Pathological processes	Count	Diseases	Count
TGF-β	853	Growth	1127	Breast Cancer	1144
Fibroblast Activation Protein	254	Metastasis	947	Colorectal Cancer	682
NF-κB	237	Progression	733	Pancreatic Cancer	502
Endothelial Growth-Factor	229	Epithelial Mesenchymal Transition	654	Lung Cancer	384
Interleukin-6	151	Invasion	590	Hepatocellular Carcinoma	309
Collagen	134	Activation	506	Prostate Cancer	292
α-SMA	134	Angiogenesis	504	Gastric Cancer	227
Caveolin-1	108	Proliferation	370	Head And Neck Cancer	207
Podoplanin	103	Migration	355	Ovarian Cancer	168
Matrix Metalloproteinases	102	Inflammation	234	Cholangiocarcinoma	102

**Figure 8:**
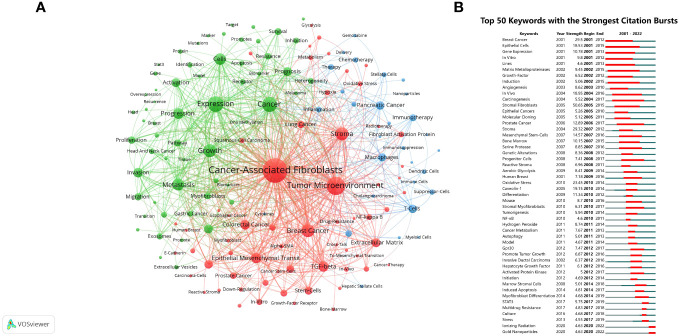
**(A)** Network map of keywords for CAFs studies. **(B)** The top 50 keywords with the strongest citation bursts.

The analysis of keyword clustering provided insights into the topic distribution within the research field of CAFs, thereby enhancing the clarity of the specific research content in this field. **Figure 8A** depicted a visual representation of the keyword network, revealing the presence of three distinct clusters. The predominant red cluster primarily encompassed the investigation of CAFs’ environmental context, their associations with specific diseases, and the exploration of relevant molecular proteins, including TME, stroma, breast cancer, colorectal cancer, TGF-β, and NF-κB. Meanwhile, the green cluster focused on the relationship between CAFs and the pathological processes of tumours, such as growth, metastasis, and progression. The blue cluster was mainly studied in relation to CAFs in relation to tumour therapy and immune cells such as immunotherapy, T cells, macrophages and dendritic cells.

The analysis of keyword bursts provided insights into popular trends in keywords and their temporal distribution, as depicted in **Figure 8B**. The keywords that experienced bursts during the early period (2001–2007) primarily included epithelial cells, matrix metalloproteinases (MMP), growth-factor, angiogenesis, and mesenchymal stem-cells. During the middle period (2008–2016), there was a notable decrease in the occurrence of keyword bursts, particularly in research pertaining to cancer pathological processes, encompassing genetic alterations, reactive stroma, oxidative stress, hydrogen peroxide, cancer metabolism, autophagy, promotion of tumour growth, and induced apoptosis. From 2017 to 2022, the predominant focus of research on CAFs pertained to cancer treatment, with particular emphasis on STAT3, multidrug resistance, ionizing radiation (IR), and gold nanoparticles (GNPs) emerging as prominent areas of investigation.

## Discussion

CAFs are the most numerous stromal cells in TME, which can not only directly alter the immunosuppressive effect of TME through their own metabolism but also influence the aggregation and function of immune cells by secreting a large number of factors, thus reducing the body’s immune surveillance of the tumour cells, and making the tumour more susceptible to immune escape (21, 22). In light of the therapeutic potential of CAFs within the TME, we conducted a bibliometric analysis using publication data from the Web of Science (WOS). We aimed to elucidate the current development trends and prominent research directions about CAFs. Based on the consistent upward trajectory observed in global publications, it is evident that CAFs have garnered significant interest among researchers, as evidenced by the substantial number of publications exceeding 1,000 in 2022. Furthermore, as of the present search, approximately 900 publications have already been released in 2023, indicating a significant ongoing research potential within the field of CAFs.

In terms of countries/regions and institutions, it could be seen that the number of publications and the number of citations were not precisely the same. Although the USA had the second-highest number of publications, it had the highest average number of citations and displayed extensive collaborative centrality with other countries/regions and institutions. Additionally, it is noteworthy that 7 out of the top 10 institutions in terms of publications were situated in the USA, thus substantiating its position as a frontrunner in CAFs research technology and its substantial contributions to the field. China recorded the highest volume of publications, with Shanghai Jiao Tong University emerging as the foremost institution in terms of publication count. However, it is noteworthy that the average citation count was relatively low, suggesting a limited degree of collaboration with other countries/regions and institutions. These findings suggest that while China has shown a significant commitment to research in the field of CAFs, with substantial investments in research personnel and funding, the quality of academic papers produced in China displays considerable variation. This is also a side effect of the fact that the size of a country’s population introduces a bias in research. The USA has a smaller population than China, so the number of studies will be smaller than China. However, one of the drawbacks of overpopulation is that it produces many low-quality articles, which can have a detrimental effect on academics. It is imperative to prioritize the quality of research papers and foster increased collaboration and cooperation with esteemed institutions in foreign countries, aiming to augment academic influence. Meanwhile, the top 10 countries/regions or organisations in terms of the number of publications were mostly in the developed European and American countries, and there was a high degree of cooperation among them, which confirmed their advanced technology and sensitivity to the research hotspots in the medical field industry, especially in the field of CAFs. It is easy to see that the wealth of different countries is also a potential reason for the existence of a research bias, as this would limit investment in health research. Developed countries have more wealth, so that research can be funded more, is more intensive and yields more results. It is also worth noting that an author may be involved in several working affiliations simultaneously, but only one may be the primary, real working institution. This will inevitably result in a less precise count of authors and institutions. This may be caused by the fact that the author is attached to more than one institution, which may be an honour, whereas there is only one main institution. Or the author changes the body of work, but the paper retains the initial body of work. One way to really solve this statistical problem is to rely on the fact that authors need to mark their main working institution when papers are reviewed. Secondly, the literature database needs to be constantly updated with algorithms that can promptly and accurately label and update the institutions where authors are located at the time of publication and limit the number of institutions to which the literature is exported. Thirdly, it is checked and verified through manual auditing by many people, but this is a very heavy workload that could perhaps be aided by artificial intelligence.

Sotgia, Federica, Martinet, Wim, and Lisanti, Michael P emerged as trailblazers and frontrunners in the domain of CAFs, exhibiting exceptional prowess in authorship and co-authorship analyses. These esteemed individuals demonstrated remarkable leadership by virtue of their substantial contributions in terms of publication count, citation count, and H-index. Their research pursuits demonstrated significant overlap, and their scholarly works primarily showcased collaborative efforts. They focused on how certain proteins and genes drive the phenotype of CAFs, as well as exploring the mechanisms by which CAFs promote tumour growth and progression through tumour metabolism, such as aerobic glycolysis and mitochondrial oxidative metabolism, and confirming the important role of oxidative stress on CAFs’ metabolism (23–30). Studies were also involved in the metabolic effects of inflammatory signals such as NF-κB with oncogenes on TME and worked on the development of anticancer drugs (31–33). Meanwhile, from the author cooperation graph, it could be found that there was active cooperation among authors from different countries/regions and institutions, but the cooperation was still dominated by the same countries/regions and institutions.

Cancers emerged as the journal with the highest number of publications, whereas Cancer Research garnered the highest number of citations. Most of the journals occupying top positions in publications and citations were situated within the JCR Q1 region, signifying their significant academic influence. Notably, Frontiers in Oncology, Frontiers in Immunology, Cancers, Oncogene, International Journal of Molecular Sciences, and Cancer Letters serve as the primary platforms for disseminating information on CAFs at present. Simultaneously, Cell, Nature, and their affiliated sub-journals emerged as preeminent publications within the domain of CAFs, garnering substantial citation counts. Consequently, researchers ought to diligently enhance the paper’s caliber in subsequent stages, with the ultimate objective of securing publication in Cell, Nature, or their associated sub-journals.

The disciplinary analysis of journals revealed that research in the field of CAFs was concentrated in the disciplines of medicine, medical, and clinical, as well as in the disciplines of molecular, biology, and immunology. Additionally, the disciplines of health, nursing, and medicine, along with the disciplines of molecular, biology, and genetics, provided a strong knowledge base for research in these fields. Publications in the molecular, biology, and immunology disciplines were mainly influenced by publications in the molecular, biology, and genetics disciplines. Combined with the bi-plot superimposition analysis, the research articles in the field of CAFs were mainly published in journals in the disciplines of molecular, biology, immunology, and medicine. Molecular, biology, immunology, medicine, and genetics were the main research disciplines in this field. Meanwhile, we can see that the research of CAFs is multidisciplinary and multidisciplinary, and the communication and mutual influence among various disciplines also help to promote the progress and development of the field.

Analysis of the references showed that 7 of the top 10 most cited articles were clinical trial articles, two reviews and one theoretical framework. These publications primarily focused on breast, pancreatic, and skin cancers, thereby reflecting the extensive research background on CAFs and carrying significant implications for the advancement of CAF-related research. Co-citation and timeline analyses can better reflect the direction of research in a particular field and the development of academic crossover, and it can be seen that the research centre of gravity of CAFs has begun to shift towards extracellular vesicle,extracellular matrix,pan-cancer analysis,single-cell atla,preclinical evaluation and so on. In terms of the literature explosion, we found that most of the early studies on CAFs were focused on breast cancer; for example, CAFs promoted breast cancer growth and angiogenesis through the secretion of SDF-1/CXCL12, and induced metastasis through the secretion of the chemokine ligand 5 (19, 34). In addition, CAFs mediated the pro-inflammatory effects of tumours through NF-κB signalling (35). In the medium term, the direction of research shifted from the cancer cells to the microenvironment in which they were embedded, such as glycolysis and interactions between CAFs of different phenotypes in the tumour stroma (36, 37). The role of CAFs in TME also received increasing attention (38, 39). At the later stage, the focus of research on CAFs was reflected in the heterogeneity of CAFs and their different phenotypes, and exploring targeting the phenotypic heterogeneity of CAFs for anti-tumour effects, as well as more in-depth research on CAFs in TME (1, 40–43). Furthermore, Pancreatic Ductal Adenocarcinoma emerged as a prominent focus in CAFs research (44, 45). However, our investigation revealed a dearth of literature on CAFs in the cancer treatment context, with limited representation of various cancer types. Consequently, it is imperative for future scholars to augment research endeavours in the realm of clinical treatment and delve into the correlation between CAFs and specific cancer types, such as the association between CAFs and lung cancer alongside pulmonary fibrosis.

From the keywords co-occurrence and clustering analyses, the research direction on CAFs shifted from the molecular biological properties of CAFs and the mechanism of CAFs in the pathological process of tumour diseases to the cancer therapy targeting CAFs. CAFs were clearly confirmed to facilitate tumour growth, metastasis and invasion, etc., and were closely associated with tumour progression, especially in breast, colorectal and pancreatic cancers.

From the keywords burst, the research direction of CAFs was roughly the same; here, we focused on analysing the molecular targets and mechanisms of action of CAFs with tumours. Angiogenesis is necessary for tumour cell proliferation and survival (46). The keyword hotspots of early CAFs were all related to angiogenesis. CAFs could secrete a large number of growth factors, cytokines and chemokines and mediate related signalling pathways such as TGF-β/TGF-β receptor, VEGF/VEGF receptor and SDF-1 (CXC12)/CXC chemokine receptor type 4, or synthesize related proteins such as podoplanin, FAP and Winglesstype MMTV integration site family member 2 (47), or secret MMP-9/13 to promote tumour angiogenesis (48–51).

At mid-term, oxidative stress had the highest citation burst intensity among keywords. Active expression of TGF-β resulted in the deletion of Caveolin-1, which led to metabolic reprogramming of CAFs and promoted their autophagy and aerobic glycolysis, resulting in oxidative stress (52). The excessive presence of oxidative stress within cells can induce modifications in DNA bases, leading to the formation of strand breaks, activation of proto-oncogenes, and inactivation of oncogenes. The progression and development of tumours are intricately linked to these molecular events. Oxidative stress in the body primarily generated reactive oxygen species (ROS), with hydrogen peroxide (H_2_O_2_) being the principal ROS produced by tumour cells. H_2_O_2_ was a major signalling molecule that could promote cancer cell proliferation, survival and invasion through redox signalling (53). Interestingly, another study showed that impaired TGF-β signalling led to higher H_2_O_2_ production by CAFs (54). This proves that our studies on CAFs are far from sufficient, and more experiments are still needed to clarify their relationship. Targeting the redox pathway seems to be a potential therapeutic strategy for targeting CAFs against tumours. Also, myofibroblast differentiation had a higher bursting intensity, which played a pivotal role in the malignant progression of lobular tumours. Tumour cells could activate mesenchymal fibroblasts, leading to their differentiation into CAFs with myofibroblast characteristics, thereby enhancing the aggressiveness of the cancer (55).

At a later stage, the investigation of cancer therapy targeting CAFs emerged as a prominent area of research. Signal transducer and activator of transcription-3 (STAT3) was identified as a critical mediator of the oncogenic effects of CAFs and was a key transcription factor regulating the function of CAFs while crosstalking with tumour cells and immune cells within the TME (56, 57). For instance, the pro-inflammatory cytokine IL-6 stimulated the expression of Twist1 in normal fibroblasts, leading to their transdifferentiation into CAFs through STAT3 phosphorylation, thereby facilitating tumour invasion (58). So, targeting STAT3 is a promising therapeutic target. Meanwhile, CAFs could provide cancer cells with protective ecological niches for anti-cancer drugs, while the symbiotic relationship between tumours and CAFs provided key resources, such as growth factors and nutrients, for optimal tumour growth and proliferation, so CAFs promoted tumour therapeutic resistance (59, 60). A study showed that drug-resistant tumour tissues from gastric cancer patients were enriched in CAFs, which promoted the development of chemoresistance by secreting IL-11 and activating the IL-11/IL-11 receptor/glycoprotein 130/Janus kinase/STAT3 anti-apoptotic signalling pathway in gastric cancer cells, thus targeting IL-11 to CAFs could alleviate drug resistance (61). In addition to resistance to cancer treatment drugs, CAFs are also involved in resistance to radiotherapy. Although IR was effective in killing cancer cells (62), CAFs could attenuate IR-induced cancer cell death by modulating the DNA damage response. Simultaneously, the activation of CAFs by IR might induce cancer cell proliferation, migration, and radiotherapy tolerance (63). In contrast, CAFs subjected to IR lost their pro-tumour growth potential *in vivo*, which might be related to affecting angiogenesis and tumour engraftment (64). IR inhibited the proliferation, migration and invasive ability of CAFs in lung cancer, which might be related to the power of IR to promote MMP-3, inhibit MMP-1, and enhance integrins α2, β1 and α5 to stabilise lesion exposure (65). It can be seen that the effect and mechanism of action of IR on CAFs are more complex and may also be related to the radiotherapy dose and changes in the secretion rate and composition of extracellular vesicles (66), which need to be further researched and investigated. Another hotspot in the keyword explosion was GNPs, which had been applied to the field of CAFs due to their unique biological functions. GNPs converted activated CAFs to a quiescent phenotype, which might be related to the endogenous synthesis of lipids induced by GNPs (67). Meanwhile, GNPs could inhibit the activation of CAFs and exert anti-CAFs effects, which might be due to their ability to alter a variety of fibroblast activating or inactivating proteins secreted by cancer cells and TME cells such as TGF-β1 and PDGF-1 (68, 69). Meanwhile, the high uptake of GNPs by CAFs in radiotherapy confirmed that GNPs could be an effective sensitiser in future cancer radiotherapy, demonstrating the very high therapeutic potential of cancer nanotechnology (70, 71).

The utilization of bibliometric analysis serves as a crucial approach for investigating the trajectory of development and identifying prominent areas of research within the domain associated with CAFs. However, it is imperative to acknowledge that this study methodology is not without its constraints. Firstly, the reliance on the WOSCC database for retrieving literature information may lead to the exclusion of pertinent data from alternative databases. Secondly, the omission of publications from the year 2023 may result in a failure to capture the most recent research hotspots and trends. Thirdly, despite employing double-checking measures, the necessity for manual correction of overlapping or inaccurate elements remains unavoidable. Fourthly, the limitations of the research algorithms of the bibliometrics themselves have allowed the contributions of many emerging researchers to be overlooked, while information bias between different measurement software has led to inconsistent results. Finally, the removal of articles other than Article and Review from the inclusion criteria may introduce some bias, although the overall bias is manageable due to the relatively small number of exclusions.

In summary, the field of research on CAFs is experiencing rapid advancements, and there is a growing anticipation that cancer therapy targeting CAFs will emerge as a prominent area of investigation. Furthermore, the next phase requires extensive international collaboration to provide new insights and directions for the ongoing exploration and advancement of CAFs.

## Data availability statement

The original contributions presented in the study are included in the article/**Supplementary Material**. Further inquiries can be directed to the corresponding authors.

## Author contributions

W-CY: Investigation, Writing – original draft, Writing – review & editing. J-XZ: Investigation, Writing – original draft, Writing – review & editing. H-BC: Investigation, Writing – original draft. YY: Investigation, Writing – review & editing. Y-PZ: Investigation, Writing – review & editing. H-LZ: Investigation, Writing – review & editing. M-HL: Conceptualization, Methodology, Supervision, Writing – review & editing. W-LQ: Conceptualization, Methodology, Supervision, Writing – review & editing. H-GZ: Conceptualization, Methodology, Supervision, Writing – review & editing.
