# Supplementary material for: A bibliometric and visual analysis of cancer-associated fibroblasts
Source: Front Immunol. 2023 Dec 19;14:1323115. doi: 10.3389/fimmu.2023.1323115 (PMC10762783; doi:10.3389/fimmu.2023.1323115)
Supplement: Supplementary file 2 [file Table_1.docx]

The search formula is : TS= ("cancer-associated fibroblast*") OR TS= ("tumour-associated fibroblast*") OR TS= ("tumor-associated fibroblast*") OR TS= ("tumor associated fibroblast*") OR TS= ("tumour associated fibroblast*") OR TS= ("cancer associated fibroblast*") OR TS= ("tumor-related fibroblast*") OR TS= ("tumor related fibroblast*") OR TS= ("carcinoma-associated fibroblast*") OR TS= ("carcinoma associated fibroblast*") OR TS= ("tumor associate fibroblast*") OR TS= ("cancer-associated myofibroblast*") OR TS= ("cancer associated myofibroblast*") OR TS= ("tumor-associated myofibroblast*") OR TS= ("tumor associated myofibroblast*") OR TS= ("tumour-associated myofibroblast*") OR TS= ("tomour associated myofibroblast*") OR TS= ("tumour associate fibroblast*") OR TS= ("cancer associate fibroblast*") OR TS= ("carcinoma associate fibroblast*") OR TS= ("cancer-related fibroblast*") OR TS= ("cancer related fibroblast*") OR TS= ("tumour-related fibroblast*") OR TS= ("tumour related fibroblast*") OR TS= ("carcinoma-related fibroblast*") OR TS= ("carcinoma related fibroblast*"). The H-index, impact factor (IF), and Journal Citation Reports (JCR) divisions of the journals were acquired from Web of Science (WOS) in a scholarly manner.
